# A Unified Framework Integrating Parent-of-Origin Effects for Association Study

**DOI:** 10.1371/journal.pone.0072208

**Published:** 2013-08-26

**Authors:** Feifei Xiao, Jianzhong Ma, Christopher I. Amos

**Affiliations:** 1 Department of Genetics, The University of Texas M. D. Anderson Cancer Center, Houston, Texas, United States of America; 2 Department of Community and Family Medicine, Geisel School of Medicine, Dartmouth College, Lebanon, New Hampshire, United States of America; Pennsylvania State University, United States of America

## Abstract

Genetic imprinting is the most well-known cause for parent-of-origin effect (POE) whereby a gene is differentially expressed depending on the parental origin of the same alleles. Genetic imprinting is related to several human disorders, including diabetes, breast cancer, alcoholism, and obesity. This phenomenon has been shown to be important for normal embryonic development in mammals. Traditional association approaches ignore this important genetic phenomenon. In this study, we generalize the natural and orthogonal interactions (NOIA) framework to allow for estimation of both main allelic effects and POEs. We develop a statistical (Stat-POE) model that has the orthogonal estimates of parameters including the POEs. We conducted simulation studies for both quantitative and qualitative traits to evaluate the performance of the statistical and functional models with different levels of POEs. Our results showed that the newly proposed Stat-POE model, which ensures orthogonality of variance components if Hardy-Weinberg Equilibrium (HWE) or equal minor and major allele frequencies is satisfied, had greater power for detecting the main allelic additive effect than a Func-POE model, which codes according to allelic substitutions, for both quantitative and qualitative traits. The power for detecting the POE was the same for the Stat-POE and Func-POE models under HWE for quantitative traits.

## Introduction

Genetic imprinting frequently affects genes during embryogenesis and is the most well-known parent-of-origin effect (POE). Imprinting causes the differential expression of genes based on the parental origin of the chromosome [1]. The alleles transmitted from the father have different levels of transcription and thus may render a different effect on the phenotype compared with the same alleles transmitted from the mother. Genetic imprinting has been shown to be important for normal embryonic development in mammals [2]. So far, approximately 200 imprinted genes have been validated or predicted in humans (http://www.geneimprint.com). Imprinted genes have been implicated in several complex human disorders, including diabetes, breast cancer, alcoholism, and obesity [3]–[6]. Kong et al. identified several variants of known imprinted genes showing significant effects on development of breast cancer, carcinoma and type II diabetes [7]. Recently, an allele in an imprinted region of chromosome 14q32 was identified to affect type I diabetes susceptibility by Wallace et al. [8].

Several statistical approaches have been developed for modeling POEs and imprinting effects. Shete et al. implemented a variance-components method for testing genetic linkage by incorporating an imprinting parameter [9]. They applied their method to data analysis of rheumatoid arthritis and gene expression data and found significant signals for linkage [10]. Gorlova et al. developed a method for QTL analysis to evaluate both total and parent-specific linkage signals based on identity-by-descent (IBD) sharing [11]. Ainsworth et al. described a methodology of family-based multinomial modeling in which POE detection is considered using mothers and their offspring [12]. Wang et al. developed an approach for testing transgenerational imprinting effects based on multiple pairs of reciprocal crosses [13]. Liu et al. proposed a random-effect model based on IBD by implementing the maximum likelihood method for linkage mapping of imprinting genes [14]. However, none of above approaches considered the advantage of orthogonality properties in the modeling of the main genetic effects along with imprinting effects in genome wide association studies.

Most traditional association approaches assume that the two alleles from the parents contribute equally to the trait, thereby ignoring the potentially important genetic phenomenon, POEs. These approaches estimate the main allelic effect, which could also be considered as the overall genetic effect, without considering POEs. Thus, it is important to develop new methods applicable to genome-wide scans that model the differential contribution of paternal and maternal alleles. It is desired that a method that allows for POE also maintain the power to detect the main allelic effect after adding one or more parameters to the model. Therefore, the proper and orthogonal decomposition of genetic variance renders this framework meaningful and useful to estimate main allelic effects along with the POE.

The natural and orthogonal interactions (NOIA) model was originally developed as a framework for estimating genetic effects for a quantitative trait and gene-gene (GxG) interactions [15]. The statistical formulation in NOIA provides an orthogonal approach for estimating genetic effects, which means the estimates of the genetic effects are not statistically correlated. As pointed out by Alvarez-Castro and Carlborg, there are two main advantages for the orthogonal models [15]. First, it makes model selection more straightforward. Second, it enables a proper variance component analysis because of uncorrelated estimates of the genetic effects. The NOIA model was extended by Ma et al. [16] for modeling gene-environment (GxE) interactions in quantitative or qualitative trait analysis. Simulation study and variance decomposition analysis were both performed to validate that the orthogonal NOIA statistical model are suitable to model the additive effect, dominant effect, and interaction effects.

Genome-wide association studies (GWASs) have achieved great success in identifying genetic susceptibility loci associated with human disorders and traits in the past decade, such as cancer, diabetes, hypertension and heart diseases [17]–[20]. However, explanation of the missing heritability of most complex or multifactorial diseases and disorders is still a challenge in the field of genetic epidemiology. The highly significant genetic markers identified via GWAS have explained only a proportion of the heritability of most human diseases [21], [22]. Genetic imprinting affects expression of genes and may explain some of the missing heritability.

In this study, we generalize the NOIA framework to incorporate POEs. We show that more disease-associated genes could be detected by incorporating POEs with orthogonal models than by using traditional models, and that the NOIA POE model would fulfill the requirement of maintaining the power to detect the main allelic effect for complex diseases when multiple loci contribute to disease risk. The orthogonality of the statistical formulation of NOIA framework is important, especially when multiple loci are contributing to the outcome. Using Kronecker product rule, our one-locus NOIA POE formulation can be easily extended to the general case of multiple loci (and environmental factors) to model general GxG/GxE interactions in the presence of imprinting effect, making NOIA a unified framework for detecting GxG/GxE interactions along with imprinting effect. Here we focus on one-locus association analysis for quantitative trait, implementing NOIA into a POE integrated framework by re-parameterization.

From the NOIA statistical model without POE (Stat-Usual) and the traditional functional model without POE (Func-Usual), we derived the formulas of several different quantitative trait association models, including a statistical POE (Stat-POE) model and a functional POE (Func-POE) model. Then, we evaluated the performance of the Stat-POE and Func-POE models. We also compared the performance of the POE models (Stat-POE and Func-POE) with that of the models without POE incorporated (Stat-Usual and Func-Usual). These studies were all performed for both a simulated quantitative trait dataset and a qualitative trait dataset. We found that the incorporation of POE into the statistical model did not affect the estimation of the main allelic effect. Moreover, the power of the statistical models with POE incorporated was higher in the presence of imprinting than that of the usual models without POE for detecting the main allelic effect for both the quantitative trait and qualitative trait. Although our methods were developed and evaluated for single locus association study, we show that they can be straightforwardly extended to gene-gene interaction or gene-environment interaction models.

## Methods

### The NOIA Model: Quantitative Trait Genetic Model without POE

We first briefly review the NOIA model without POE detecting. Using the usual approach for genotype-phenotype mapping of a quantitative trait locus (QTL), if the trait is influenced by a single diallelic locus, with alleles 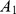 and 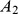, we let minor allele be 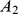. Assume we have a collected sample with 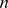 individuals. For the 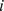-th individual, let 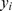 be the observed trait phenotype and 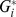 be the genotypic value for a specific locus. We use 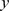
to denote the vector of the observed trait which is assumed to be normally distributed in large sample and 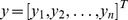. We model the phenotype as 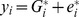. The vector 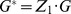, where 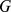 denotes the vector of genotypic values including 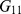, 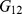 and 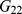 as the genotypic values of the three possible genotypes for alleles 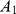 and 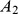; the 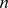 rows of matrix 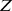 represent the corresponding genotype for 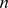 individuals. Therefore, the vector 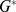 could be expressed as
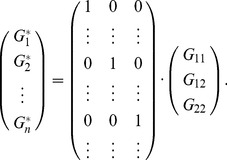
(1)


Several methods have been proposed for mapping a quantitative trait controlled by one locus with two alleles. The vector of genotypic values 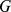 can be expressed as the product of genetic-effect design matrix 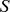 and the vector of genetic effect 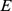.
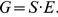
(2)


The vector of genetic effects (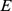) includes three parameters: the reference point (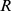), the additive effect (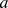) and the dominant effect (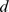).

One of the traditional regression models, which we call a functional model, is given by:
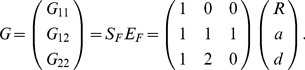
(3)


The inverse is
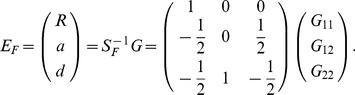
(4)


Here, the reference point 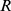 corresponds to the genotypic value of one of the two homozygotes, 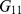. The additive effect, 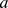, is half of the difference between the two homozygotes genotypic values. The dominance effect, 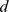, is the difference of the heterozygote genotypic value and the average of the homozygotes genotypic values. This is referred to as the Func-Usual model in what follows. Another usual functional model codes the additive effect as (−1,0,1) for the three genotypes and the reference point corresponds to the average genotypic values of the two homozygotes [23]. These two usual functional models have the same estimators except the intercept term, and we therefore will not discuss the second model in detail in what follows. These models are called functional models since they use natural effects of allele substitutions as parameters, mainly focusing on the biological properties [15].

A second approach to modeling, the “statistical model” which we call the Stat-Usual model, was proposed by Alvarez-Castro and Carlborg for estimating genetic effects for a quantitative trait and gene-gene (GxG) interactions [15]. It can be formulized as follows [14]:
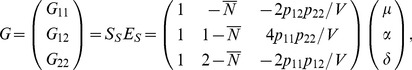
(5)which ensures the orthogonality of the estimated parameters. Here, 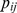 denotes the genotype frequencies of this locus in the population, where 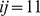, 12 or 22, and 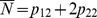, 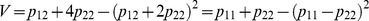. 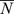 is the expected value of 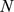 and 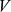 is the variance of 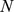, where 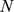 is the number of variant alleles (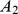) which is equal to 0, 1 or 2 when the genotype is 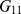, 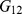 or 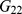, respectively. In addition, we let



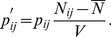
(6)The inverse of equation (5) is
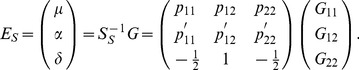
(7)


The genetic effects, 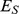, are based on the genotype frequencies of this locus in the population. Alvarez-Castro et al. [15] noted that the statistical model is an orthogonal model that has uncorrelated estimates of the parameters, which was also reflected by variance components decomposition in Ma et al. [16]. As they stated, these two models could be transformed to each other as follows:
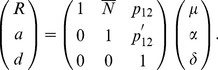
(8)


We notice the Stat-Usual and Func-Usual models have same estimators for the dominant effect and different estimators for the additive effect as 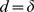. If the dominance components are removed from these two models, they have same estimation and also the same test statistic for additive effect estimation (Text S5).

### Quantitative Trait Genetic Models with POE

In this section, we extend the models described above by incorporating the POE and evaluate the performance of these extended models in detecting both the overall genetic effect and POE. For a gene with POE, the vector of genotypic values 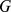 has four components, 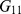,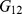,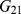 and 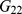, in which the first allele represented by the first digit in the subscript is transmitted from the mother, and the second allele represented by the second digit is transmitted from the father. We used 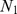 and 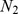 to denote the number of maternal and paternal variant allele 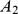, respectively. 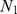 and 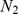 are independent variables with binomial distributions, respectively. That is,
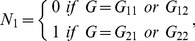
(9a)

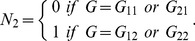
(9b)


Similar to equation (1), the vector 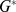 can be expressed as 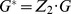 and
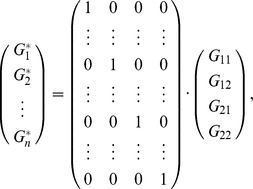
where the new 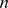 rows of matrix 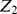 represent the corresponding genotypes for each individual.

We have two different ways to construct a functional and a statistical model with POE. First, we can do so by decomposing the additive effects into two paternal and maternal additive effects, resulting in Model 1 for the functional model and Model 2 for the statistical model (see Text S1). In this way, we are able to incorporate POE detection to the Stat-Usual model while still maintaining its orthogonality. An alternative extension of the models yields an equivalent but more comprehensive framework, which can be readily used for detecting the main allelic additive effect and POE simultaneously. The main allelic effects denote the overall additive effect on the trait conferred by this allele, and the POE is defined as the imprinting effect of the allele with paternal origin over the same allele with maternal origin. We depict these models in the following subsections and leave the details to Text S1.

### The POE Functional (Func-POE) Model

Let 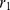 and 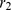 be the main allelic additive effect and POE of the locus, respectively. The functional model can be expressed as follows,
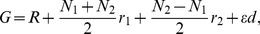
(10)or



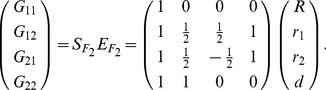
(11)The inverse of this expression is
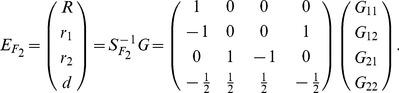
(12)


### The POE Statistical (Stat-POE) Model

Let 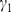 and 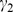 denote the main allelic additive effect and POE of the locus, respectively. Similarly, the orthogonal statistical model similar to equation (5) can be expressed as
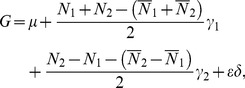
(13)where 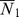 and 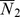 denote the means of 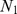 and 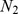, respectively, 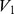 and 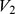 denote the variance of 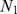 and 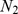, respectively. In the original models without POE (Func-Usual and Stat-Usual), 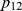 is the probability of an allele that has an allele 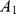 from either parent. In our new models, the meaning of 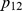 is different: it is the probability of an allele that has allele 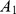 from the mother and allele 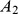 from the father. Similarly, 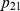 is the probability of an allele that has allele 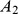 from the mother and allele 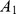 from the father. From equations (9a) and (9b), we have



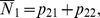


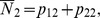


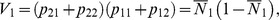


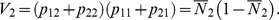



Therefore, according to equation (13), the vector of genotypic values can be expressed as
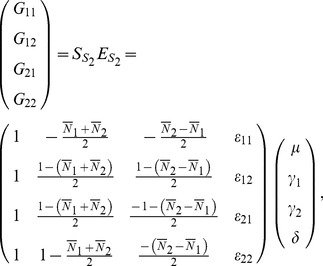
(14)where
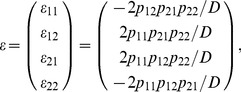
(15)and



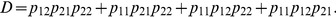
(16)The inverse is then 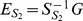, which can be expressed as
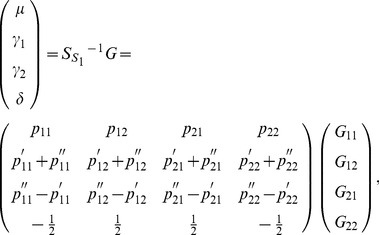
(17)where



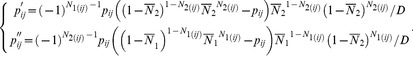
(18)

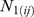
 and 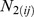 denoted the 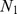 and 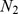 values of the genotype 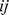, respectively. From equations (17) and (18), each column of 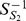 is independent of the others therefore the parameters are orthogonal.


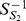
 can also be expressed as
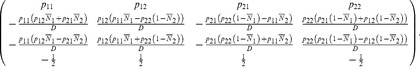
(19)


The POE functional model (Func-POE) and statistical model (Stat-POE) are related by
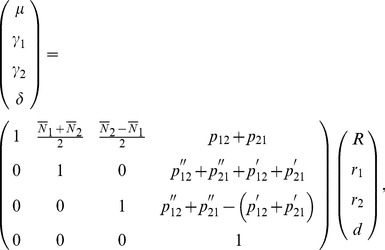
(20)where



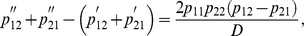
which means 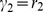 in the case of equal frequency of the two types of heterozygote (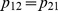).

### Orthogonality of the Stat-POE Model

We have previously showed that the Stat-Usual model was orthogonal in the sense that the estimates of the four parameters were uncorrelated [16]. As stated in the previous section, from equations (17) and (18), the lack of correlation of the column values of 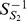 implies that the Stat-POE model is also orthogonal. The fact that the variance of 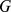 can be decomposed into two independent additive components and one dominant component also reflect the orthogonality of the statistical imprinting model. To prove the orthogonality, we start with equation (13) to decompose the total genetic variance as follows
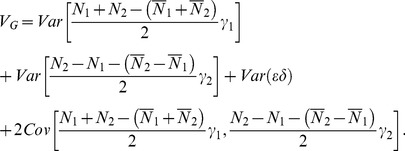
(21)


Note that
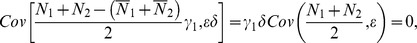
and similarly,



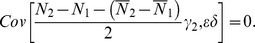



Also, 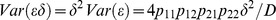. Therefore, we could express the additive and dominant variance components as
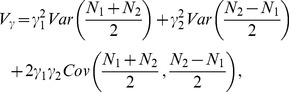
(22)

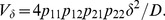
(23)


To show that the additive variance, 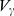 , could be decomposed to be two parts that are dependent on only two additive effects (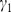 and 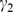) respectively, 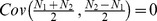 needs to be satisfied. And, as we know
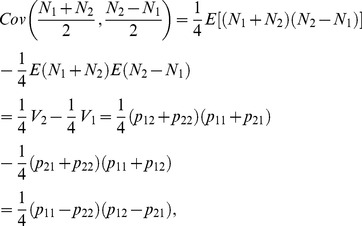
(24)which is indeed equal to 0 under the condition that 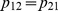 or 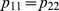. In this way, we divided the additive variance component into two independent parts as follows:



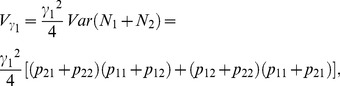
(25)

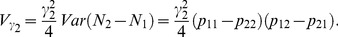
(26)


And 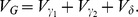


The two additive variance components 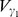 and 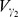 are related only to the additive effects parameters 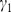 and 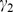, representing the overall genetic effect and the POEs, respectively. The dominance variance component 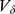 is only related to the dominance effect parameter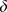. The property that the variance components can be divided into two independent additive components and one dominant component demonstrates that the transformed POE statistical model is orthogonal. We also proved that the Stat-POE model is orthogonal before transformation (Text S2). On the other hand, by checking whether 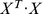 is a diagonal matrix, we showed that the transformed Stat-POE model is orthogonal (Text S3). However, for the transformed Func-POE model, the variance components could not be decomposed into three independent parts, indicating that the Func-POE model is not orthogonal (Text S4).

### Simulation Methods

We performed simulation studies for both a quantitative trait and a qualitative trait (case-control) using an approach similar to that used in [16], and the simulated data were analyzed using the four aforementioned models: Stat-POE, Func-POE, Stat-Usual and Func-Usual. The Wald test was used to test the hypothesis that the corresponding coefficient of the genetic effects and imprinting effect was equal to 0. The test statistic was 
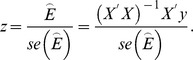
 The R coding for the Stat-POE and Func-POE models is available in Text S6.

#### Simulation of data with a quantitative trait

To simulate samples of independent individuals with a quantitative trait controlled by a diallelic locus, we assumed that the gene is under HWE. The case that a gene is not under HWE was not considered in our study, and will be investigated in our future work. For a given value of the minor allelic frequency (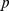) in the population, genotype 11, 12, 21, 22 were assigned to an individual with probabilities 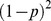, 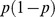, 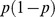 and 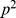 respectively. We assumed the genotype frequencies of the two types of heterozygotes were the same in the population. We assumed the phenotype was influenced by a main allelic additive effect, a POE, and a dominant effect. From a prespecified vector of parameters (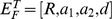), we assigned each individual a genotypic value according to his/her assigned genotypes. Then, by randomly generating a value from a normal distribution with prespecified mean and variance (0 and 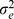), we generated an observed phenotype/trait by adding this residual to the previously assigned genotypic value. We used data from 2000 individuals as a replicate and simulated 1000 replicates for each genetic model.

In the simulation study of a quantitative trait, three scenarios were simulated with different levels of POE (Table 1). The minor allele frequency (MAF) 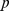 was set to 0.28, and the residual variance 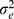 was 144.0. The true values of the four parameters in these three scenarios are shown in Table 1. For the sample size with 2000 individuals, the computation speeds of the four models running by R programming in Unix system are: 22 seconds for the Stat-POE model, the Stat-Usual model and Func-Usual model, respectively; 24 seconds for the Func-POE model.

**Table 1:** Simulation true values of genetic effects for quantitative and qualitative traits data sets.

	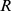	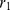	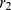	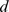
**Quantitative trait**				
Scenario 1	90.0	3.0	−3.0	1.2
Scenario 2	90.0	3.0	−2.0	1.2
Scenario 3	90.0	3.0	−1.0	1.2
**Qualitative trait**				
Scenario 1	100.0	2.0	−2.0	0.5
Scenario 2	100.0	2.0	−0.6	0.5

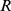
 denotes intercept; 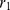 and 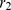 denote overall additive genetic effect and POE, respectively; and 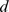 denotes dominant effect. Three scenarios with strong, medium and weak POE were simulated for quantitative traits; two scenarios with strong and weak POE were simulated for qualitative traits. The MAF was set to 0.28 for both traits.

#### Simulation of data with a qualitative trait

Ma et al. [16] derived the formulation of the statistical model without POE incorporated in quantitative traits and proposed that a similar statistical model could also be defined for a qualitative trait by treating the genetic effects as the logit function of the disease. Unfortunately, the orthogonality of that model is not valid for the qualitative trait under the alternate hypothesis that there is a genetic effect, but is valid under the null hypothesis of no effect. This same conclusion is true for our POE statistical model. Here we performed simulations to evaluate the performance of the POE-related models in a case-control study design.

Briefly, we used the logistic model and Bayes’ theorem to set the genotype of each individual according to the prespecified genetic effect terms, 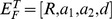. The disease penetrance for each genotype was determined by
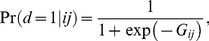
(27)where 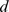 denotes the disease status with value 1 for cases and 0 for non-affected controls.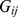 was the genotypic value when the genotype was 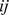 with 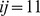, 12, 21 or 22. Then the distribution of the four genotypes in the cases was determined by



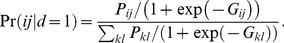
(28)As in the simulation study for a quantitative trait, 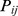 is the genotype frequency of 11, 12, 21 and 22 in the population, determined by 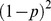, 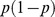, 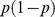 and 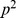, respectively. For simulating controls in the population, we used a similar distribution as follows
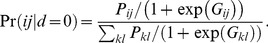
(29)


For each replicate, 1000 cases and 1000 controls were generated, and a total of 1000 replicates were simulated. The MAF 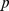 was set to 0.28. Two scenarios were simulated with different levels of POE (Table 1).The simulating values of the parameters in the two different scenarios are shown in Table 1.

To determine whether the setting of the MAF value influence the performance of the models, we also simulated two additional scenarios with different MAF values (0.03 and 0.48) for both quantitative traits and qualitative traits.

## Results

**Figure 1:**
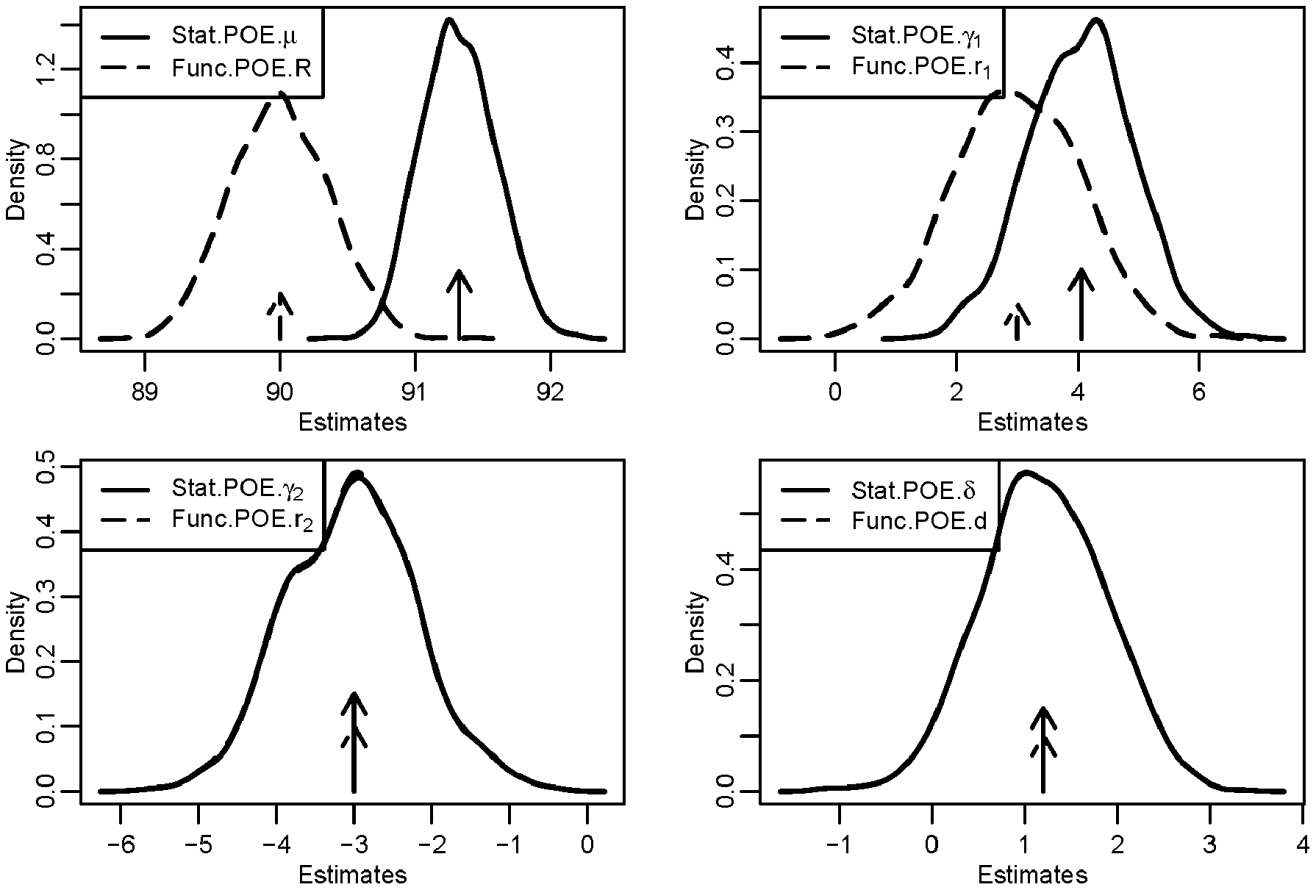
Density distribution of the estimates of the parameters from a simulated data analysis with a quantitative trait influenced by a genetic factor and by strong POE (Scenario 1). The pre-specified minor allele frequency was 0.28. The values of the four parameters were 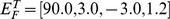 and 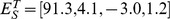 for the functional POE (Func-POE) model and the statistical POE (Stat-POE) model, respectively. The solid arrows denote the true simulated values of the parameters for Stat-POE model and the dashed arrows denote those for the Func-POE model.

First we performed a simulation study for a quantitative trait in three scenarios with strong, moderate, or weak imprinting effect while the main allelic additive effect remained the same (Table 1). The true values of the four parameters in these three scenarios are shown in Table 1. The density distribution of all four effects after analyzing 1000 replicates in scenario 1 with strong imprinting effect is shown in Figure 1. The estimates of all four parameters were accurate for both the Stat-POE and Func-POE models. Compared with the Func-POE model, the Stat-POE model had smaller variance in most cases for detecting the intercept and main allelic additive effect terms. The estimates for the POE term and dominant effect term were the same between the Func-POE and Stat-POE models. Similar patterns could be detected for the other two scenarios (data not shown).

**Figure 2:**
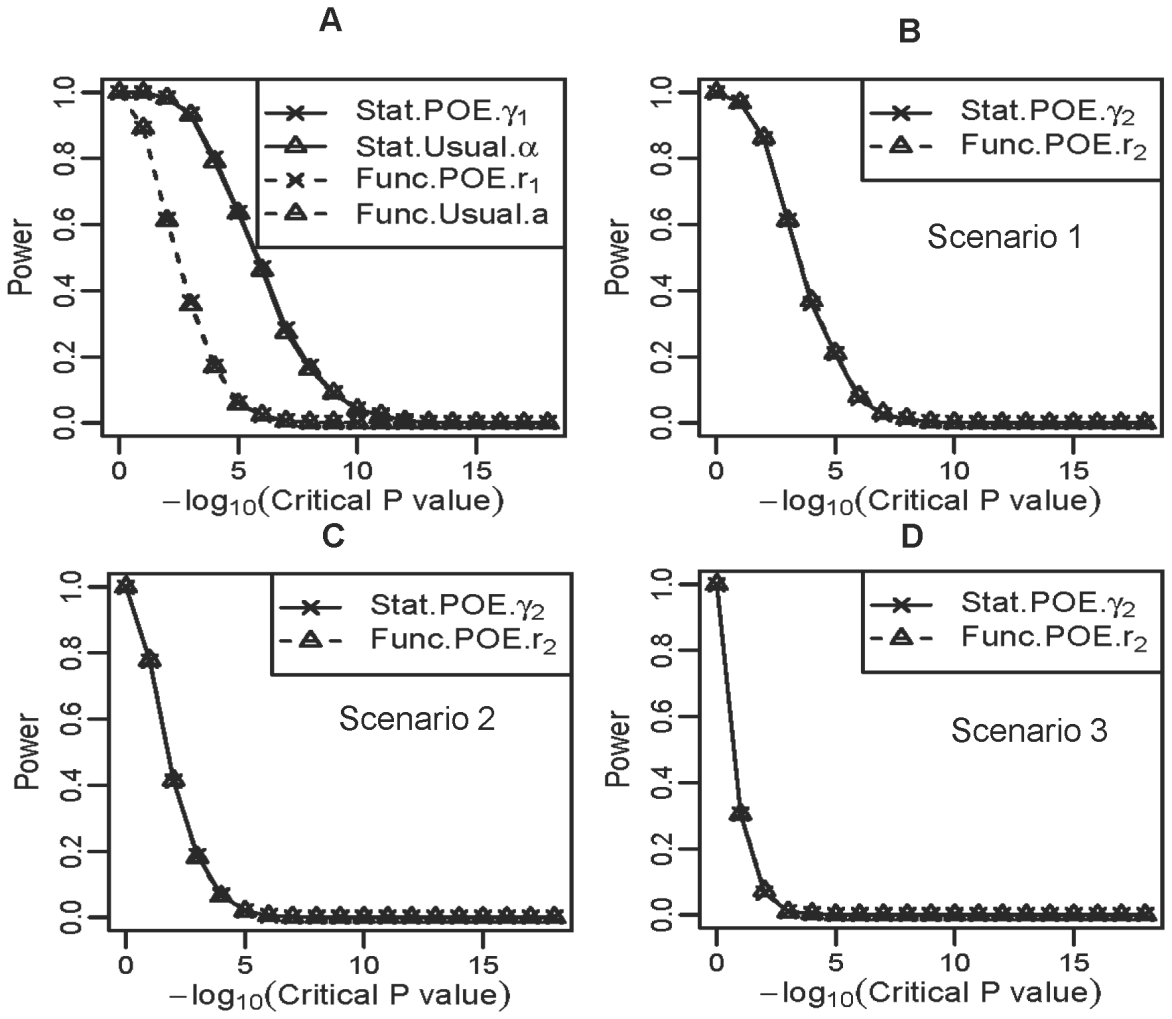
Power under different critical values of the P values obtained using the Wald test for the quantitative simulation data shown in Table 1. (a) Power for detecting the main allelic additive effect in scenario 1 when strong POE exists. Power for detecting POE of the Stat-POE and Func-POE models was compared for scenario 1 (b), scenario 2 (c), and scenario 3 (d).

To evaluate the performance of these models in detecting main allelic additive effect and POE, we calculated the statistical power of four models under different critical values of P values obtained using a Wald test (Fig. 2). Figure 2a shows the power for detecting the main allelic additive effect for scenario 1 with strong POE. The power of both statistical models (Stat-POE and Stat-Usual) for detecting additive effects was greater than that of both functional models (Func-POE and Func-Usual). The power of detecting additive effect was the same for the Stat-POE and Stat-Usual models. It was also the same for the Func-POE and Func-Usual models. In the other two scenarios in which medium or weak POE was simulated, identical results were obtained for the main genetic effect term as shown in Figure 2a, since the main allelic additive effect was set to the same value, 3.0 (Table 1). These results indicated that the power for detecting the main allelic effect did not change even if a POE parameter was integrated into the analysis model. The performance of these four models for detecting dominant effects was the same in three scenarios (data not shown), which was consistent with the formulations (Equation (8) and Text S5).

Figure 2b–d shows the power of the Stat-POE and Func-POE models for detecting the POE in three scenarios. The performance of the two POE models remained the same in scenarios 1, 2 and 3. This is because in our simulation, the genotype frequency values for the two types of heterozygotes were set at the same value which is valid under HWE. We therefore have: 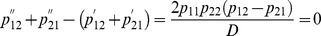. According to equation (20), the POE repressor in the Stat-POE model was equivalent to that in the Func-POE model. When the assumption that the genotype frequencies for the two heterozygotes are the same is violated, we would see different performance for the Stat-POE and Func-POE models in detecting POE (data not shown). We also found that the overall power decreases when the POE decreases (Fig. 2b–d).

To evaluate whether the MAF influences the estimation of the genetic effects by these models, we also performed analyses for quantitative traits when the MAF was 0.03 and 0.48, respectively (Fig. S1–S2). Figure S1 shows that when strong POE existed, the Stat-POE model still had much greater power than the Func-POE model in detecting the main additive effect for uncommon variants (MAF = 0.03). Figure S2 shows that when strong POE existed, the Stat-POE model had slightly greater power than the Func-POE model in detecting the main additive effect for variants with MAF as 0.48.

**Figure 3:**
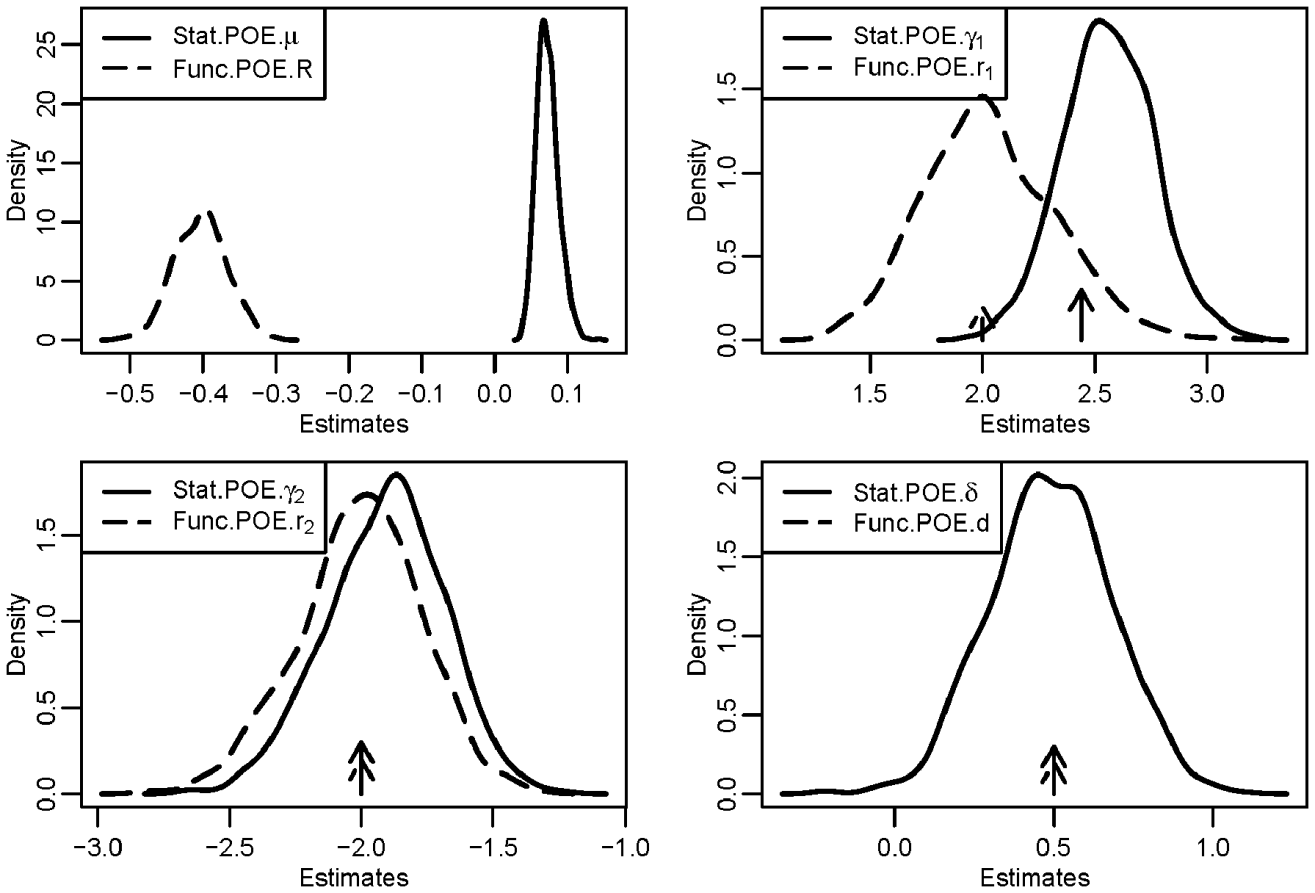
Density distribution of the estimates of all four parameters from a simulated data analysis with a qualitative trait influenced by a genetic factor and by strong POE. The pre-specified minor allele frequency was 0.28; the true values of the four parameters were 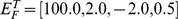 and 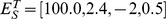 for the Func-POE and the Stat-POE models, respectively. The solid arrows denote the true simulated values of the parameters for Stat-POE model and the dashed arrows denote those for the Func-POE model.

Similarly, we also performed analyses for simulated case-control data. The simulating values for each of the two scenarios are shown in Table 1. Figure 3 shows the histograms for all four effects after analyzing 1000 replicates in scenario 1. The patterns for the distribution of the four parameters were similar to those observed in the simulation of a quantitative trait, and the estimates were very close to the corresponding simulating values for both the Stat-POE and Func-POE models, except for the intercept term. The differential estimation of the intercept term arose from the non-random sampling in our simulation: the proportion of cases in the sample was much larger than that in the general population. The variance of the main allelic additive effect for the Stat-POE model was still smaller than that in the Func-POE model. The estimate distributions are very close to each other or the same for these two models in detecting POE and dominant effect.

**Figure 4:**
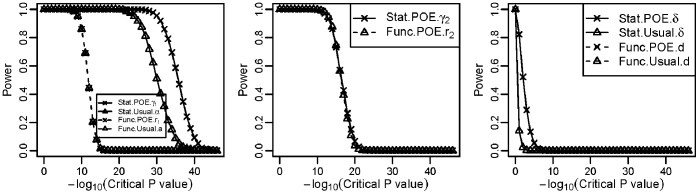
Power under different critical values of the P values obtained using the Wald test for the case-control simulation data influenced by a genetic factor with strong POE (scenario 1). The minor allele frequency was 0.28.

Figure 4 shows the power of the four models in detecting the main allelic additive effect, POE and dominant effect when the trait was affected by relatively strong POE for case-control data. The performance of the Stat-POE model was slightly better than that of the Stat-Usual model, but the performance of both was better than that of the functional models, Func-POE and Func-Usual (Fig. 4a). The Stat-POE and Func-POE models had the same power in detecting POE (Fig. 4b, 4c). Interestingly, the POE models (Stat-POE and Func-POE) both had higher power for detecting dominance effect than the usual models, Stat-Usual and Func-Usual (Fig. 4c).

**Figure 5:**
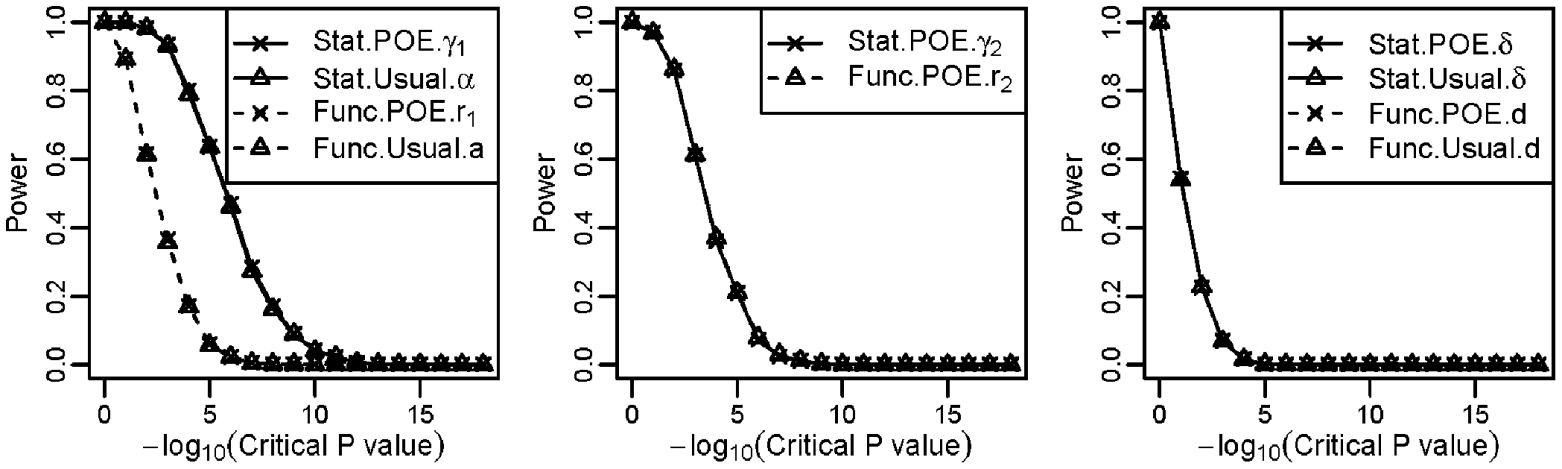
Power under different critical values of the P values obtained using the Wald test for the case-control simulation data influence by a genetic factor with moderate POE (scenario 2). The minor allele frequency was 0.28.

Another simulation was performed with a moderate POE for case-control data (Table 1, scenario 2; Fig. 5). Interestingly, the performance of the Stat-POE model was not much better than that of the Stat-Usual model (Fig. 5a) for detecting the main allelic additive effect (Fig. 4a). For detecting the main allelic additive effects, the statistical models (Stat-POE and Stat-Usual) had much higher power than the functional models, Func-POE and Func-Usual. The statistical models and functional models had the same or very close power with and without the incorporation of POE. The Stat-POE and Func-POE models had the same or very close power for detecting POE and dominant effect (Fig. 5b, 5c).

Simulations were also performed when MAF was set as 0.03 and 0.48 for case-control design, respectively (Fig. S3–S4). For rare variants, the Stat-POE model had much greater power than the Func-POE model in detecting main additive effect, although slightly greater power was observed for Func-POE model in detecting the POE (Fig. S3). For variants with MAF = 0.48, the Stat-POE model had much greater power than the Func-POE model in detecting main additive effect and dominant effect (Fig. S4). The power of the Stat-POE model was even higher than that of the Stat-Usual model in detecting the main additive effect (Fig. S4a).

We summarized the detailed power comparison of the Stat-POE and Func-POE models in Table 2. In all scenarios we simulated, the Stat-POE model had much greater (or equal) power than the Func-POE model in detecting the main additive effect. For testing imprinting effect, the Stat-POE model had the same power as the Func-POE model for quantitative traits whereas the Stat-POE model sometimes presented slightly worse power than the latter for qualitative traits. They have save performance for detecting dominant effect for both quantitative traits and qualitative traits (Table 2).

**Table 2:** Summary of the power of the Stat-POE and Func-POE models in different simulation scenarios for both quantitative traits and case-control traits.

		MAF = 0.03			MAF = 0.28		MAF = 0.48	
		Strong POE		Weak POE	Strong POE	Weak POE	Strong POE	Weak POE
**Quantitative traits**								
**Add**	**Stat-POE**	**0.98**		**0.98**	**0.93**	**0.94**	**0.79**	**0.78**
	**Func-POE**	0.01		0.01	0.36	0.35	0.75	0.75
**POE**	**Stat-POE**	0.4		0.1	0.61	0.1	0.77	0.03
	**Func-POE**	0.4		0.1	0.61	0.1	0.77	0.02
**Dom**	**Stat-POE**	0.005		0.007	0.07	0.06	0.12	0.15
	**Func-POE**	0.005		0.007	0.07	0.06	0.12	0.15
**Case-Control traits**								
**Add**	**Stat-POE**		**0.73**	**0.73**	1	1	1	1
	**Func-POE**		0.001	0.001	1	1	1	1
**POE**	**Stat-POE**		0.8	0.33	1	0.33	1	0.61
	**Func-POE**		**1**	**0.41**	1	**0.38**	1	**0.73**
**Dom**	**Stat-POE**		0.04	0.05	0.29	0.35	0.8	0.9
	**Func-POE**		0.04	0.05	0.29	0.35	0.8	0.9

Add: overall genetic additive effect; Dom = dominant effect. Threshold of the P value was 0.001.

Type I error was evaluated for both the quantitative trait and the qualitative trait by simulating a null scenario where there was no main genetic effect or POE. We estimated the type I error for the main additive effect, POE and dominant effect for both quantitative traits and case-control traits when the MAF was set as 0.03, 0.28 or 0.48 (Table 3). The false positive rate for detecting the additive effect was almost the same for the statistical and functional POE models in most scenarios we simulated (around 0.05 or less for the 1000 replicates). The false positive rate for detecting the additive effect was smaller estimated from the Func-POE model than that from the Stat-POE model, when MAF was set as 0.03 for case-control traits. For detecting POE, these two models usually had very close false positive rates for both quantitative and case-control traits.

**Table 3:** Type I error for simulation of quantitative and case-control traits data sets.

	MAF = 0.03			MAF = 0.28			MAF = 0.48		
Models/MAF	Add	POE	Dom	Add	POE	Dom	Add	POE	Dom
**Quantitative trait**									
**Stat-POE**	**0.047**	**0.037**	**0.059**	**0.055**	**0.038**	**0.048**	**0.053**	**0.043**	**0.043**
**Func-POE**	0.055	0.036	0.059	0.056	0.037	0.048	0.052	0.042	0.043
**Stat-Usual**	0.048		0.06	0.056		0.048	0.053		0.044
**Func-Usual**	0.048		0.06	0.056		0.048	0.053		0.044
**Case-control trait**									
**Stat-POE**	**0.044**	**0.062**	**0.017**	**0.05**	**0.045**	**0.046**	**0.047**	**0.049**	**0.039**
**Func-POE**	0.01	0.063	0.017	0.049	0.047	0.046	0.049	0.048	0.039
**Stat-Usual**	0.045		0.017	0.047		0.047	0.047		0.038
**Func-Usual**	0.045		0.017	0.047		0.047	0.047		0.038

False positive rates for the genetic effects estimated from the Stat-POE, Func-POE, Stat-Usual and Func-Usual models under different minor allele frequency settings. **Add** = overall genetic additive effect; Dom = dominant effect; MAF = minor allele frequency.

## Discussion

In this study, we extended the NOIA framework, which was initially developed for epistatic analysis of quantitative traits, by incorporating POE for genetic association analysis. Herein, we propose a unified framework for one-locus association study that allows for main allelic additive effect, the dominant effect and POE estimation via linear regression or logistic regression. Using simulation, we illustrated the statistical properties of this extended framework on one-locus association study. Although the Func-POE model sometimes presented slightly greater power than the Stat-POE model for estimation of POE for qualitative trait. The Stat-POE model are always preferred than the Func-POE model in detecting overall additive effect for quantitative traits and qualitative traits, because of its much greater power.

We conducted genetic variance decomposition to show that the Stat-POE model was orthogonal when either HWE or equal minor and major allele frequencies is satisfied for quantitative traits (equations 21–25). Thus, even when the POE was absent, estimates of the overall genetic effects were not affected after a new parameter representing POE was added in the analytic model. This was not true for the Func-POE model, as shown in our simulation for quantitative traits (Fig. 2a). Although the Func-POE model was not orthogonal (Text S4), we have the same performance of the Func-POE and Func-Usual models for detecting the main allelic additive effect, as shown in Figure 2a. This is probably because the term 
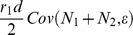
 in equation (D3) (Text S4) is rather small in our simulation. The Stat-POE and Func-POE models we proposed could also be applied to qualitative traits via logistic regression although the property of orthogonality would no longer exist under the alternative model [16]. When orthogonality exists under the null, the subsequent tests have appropriate type I error rates, but the failure of orthogonality under the alternate model can lead to improper estimates of heritability, although the estimators may be less biased than those that are obtained from the functional models.

Using simulation, we demonstrated that the statistical models, including the Stat-POE and Stat-Usual models, had better performance for detecting the main allelic additive effect than the functional models, Func-POE model and Func-Usual for both quantitative traits and qualitative traits. These two POE models on detecting the POE had the same power when 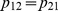. Stat-POE model had better performance on detecting the main allelic additive effect than the Stat-Usual model for qualitative traits when strong POE exists. The power was the same for detecting the main allelic effect even if a POE parameter was integrated into the analysis model because of the orthogonality of the Stat-POE model (Fig. 2a; Fig. S1–2). The performance of our framework was not exactly the same in quantitative and qualitative trait simulation studies. The simulation study for both quantitative and qualitative traits showed that the estimates of all four parameters were accurate for both the Func-POE and Stat-POE models.

However, the performance of these two models for detecting the main allelic effect and dominance effect had a different pattern in qualitative traits (Fig. 4 and 5). In qualitative traits, for detecting the main allelic effect, the statistical (Stat-POE and Stat-Usual) models, still had greater power than did the functional (Stat-Usual and Func-Usual) models in most cases, regardless of the size of the POE, which is consistent with the findings of the quantitative traits simulation study (Fig. 2). However, the power of the Stat-POE and Stat-Usual models was not usually the same for the qualitative trait simulation in different scenarios (Fig. 4a, 5a), which varied, as shown in the simulation study of a quantitative trait (Fig. 2). The performance of the four models on detecting the dominance effect was also different in the simulation of a qualitative trait (Fig. 4c; Fig. S3–4), that the POE models (including the Stat-POE and Func-POE) usually had greater power than the usual models. This difference in performance arises because the test statistics used for logistic and linear regression differ.

We also illustrate why the proposed model can detect more disease-associated genes than the traditional models in model setting as follows. First, the orthogonal (Stat-Usual) model proposed by Alvarez-Castro et al. orthogonalizes the estimation of the additive and dominant effects but the usual model (Func-Usual) does not. We constructed the test statistics of the Stat-Usual and Func-Usual models for quantitative traits with and without dominance components effects (Text S5). The test statistic for the additive effect did not change if the dominance component was removed from the Stat-Usual model. However, the test statistic was not consistent if the dominance component was removed from the Func-Usual model. Thus, we suggest that the Stat-Usual model is preferred to the Func-Usual model in association studies when a dominance component is incorporated. Second, we also compared the test statistic of the Stat-Usual and our newly developed Stat-POE models. We found that the test statistic of the main additive effect was the same for the two models, which was consistent with the simulation studies. Even in simulation studies of a case-control design, we observed that the Stat-POE had greater power for detecting the main additive effect than the usual orthogonal model (Stat-Usual). Comparing the test statistic of the Func-POE and Fun-Usual models, the estimation of the main genetic effect was not consistent, and the power decreased when POE testing was included. Therefore, Stat-POE model can detect more significant additive effect signals than the Func-POE model.

Several recent studies have incorporated POEs in association analyses for quantitative traits. Genome-wide rapid association using mixed model and regression (GRAMMAR) and its extension are a recently developed approach that is based on a measured genotype approach and has been shown to have greater power than the transmission disequilibrium test (TDT)-based tests [24]. A maximum likelihood test was also developed for detecting POEs using haplotypes [25]. Ainsworth et al. also described an implementation of a family-based multinomial modeling approach that allows for imprinting detection [12]. This method used family data, case-mother duos or case-parents trios, to look for departures in observed genotypes distribution from expected distribution among affected offspring, given the genotypes of their parents. The mechanism of analysis is still closely related to the TDT test. To our knowledge, our approach is the only one that has the advantage of orthogonality on the effects estimation for association studies of detecting POE. NOIA was previously proposed and formularized for gene-gene interaction analysis models of quantitative traits and was further implemented and extended by Ma et al. [16] to reduced genetic models and estimating effects from both genetic and binary environmental exposure. However, none of these models had the potential to detect POE. Ma et al. showed that when POE was not incorporated, the power of the statistical model (Stat-Usual) was greater than that of the functional model (Func-Usual) in most cases. This is the case in our study for detecting main effects even when POE was integrated. Our study exemplifies another significant implementation of NOIA that adopts the orthogonal property of the statistical model if the family data are available or if phasing is plausible for obtaining the parental transmitting status of the candidate disease associated loci. Because alleles of different parental origins can exert different effects, the effect contributing to the disease outcome may be masked in usual models that can detect only the main allelic additive effect. The methodology proposed here yielded a plausible means of detecting more genes that contribute to complex diseases or quantitative traits that were not detected in routine GWASs.

Although our extension of the NOIA is expected to provide new insights into disease gene mapping, pedigree data are needed for our framework to be used to estimate transmitting information of each heterozygotes or homozygotes locus. Obtaining the transmitting status of one locus is possible for non-informative pedigrees determined by nearby linked loci or haplotype phasing. Or we could use weighted analysis in which the probabilities of each genotype are used. A future direction of our next step will be to extend our formulations to incorporate non-deterministic genotypes due to insufficient parental information or missing data. Alvarez-Castro and Carlborg handled internal mapping by implementing the Haley-Knott Regression with NOIA [26]. Unknown genotypes and genotype frequencies can be estimated by the weighted computation based on the genotype at the flanking markers. The same strategy could be applied to the non-deterministic parental information and missing data.

The motivation of our implemented framework was based on the orthogonality property of NOIA which allows for easy model selection and variance component analysis. A next step is to extend the formulation proposed here to multi-locus and/or environmental factor cases, including gene-gene interaction and gene-environment interaction analyses when POE is incorporated. Conceptually, this generalization should be fairly straightforward using the Kronecker product rule as in [15], if we assume linkage equilibrium between loci and no association between a genetic locus and an environment factor. For the functional model we anticipate nonorthogonality of the estimators will result in further loss in power of hypothesis tests compared to the orthogonal tests we are proposing. However, it would probably be challenging to deal with and to properly interpret a large number of interaction terms, especially if more than two loci are involved. The extension, nevertheless, would be attractive as imprinting effects of one locus may indeed have complex interaction with main effects of other loci. We are currently working along this direction.

## Supporting Information

Figure S1 **Power under different critical values of the P values obtained using the Wald test for the quantitative simulation data influence by a genetic factor with strong POE (scenario 1).** The minor allele frequency was 0.03. Power for detecting (a) the main allelic additive effect, (b) the POE and (c) the dominant effect.
(TIFF)

Figure S2 **Power under different critical values of the P values obtained using the Wald test for the quantitative simulation data influence by a genetic factor with strong POE (scenario 1).** The minor allele frequency was 0.48. Power for detecting (a) the main allelic additive effect, (b) the POE and (c) the dominant effect.
(TIFF)

Figure S3 **Power under different critical values of the P values obtained using the Wald test for the case-control simulation data influence by a genetic factor with POE (scenario 2).** The minor allele frequency was 0.03. Power for detecting (a) the main allelic additive effect, (b) the POE and (c) the dominant effect.
(TIF)

Figure S4 **Power under different critical values of the P values obtained using the Wald test for the case-control simulation data influence by a genetic factor with POE (scenario 2).** The minor allele frequency was 0.48. Power for detecting (a) the main allelic additive effect, (b) the POE and (c) the dominant effect.
(TIF)

Text S1 **POE models before transformation.**
(DOCX)

Text S2 **Orthogonality of the Stat-POE model before transformation.**
(DOCX)

Text S3 **Orthogonality of the Stat-POE model after transformation.**
(DOCX)

Text S4 **Orthogonality of the Func-POE models.**
(DOCX)

Text S5 **Test Statistics for Full and Reduced One-Locus Models.**
(DOCX)

Text S6 **R coding of Stat-POE and Func-POE models.**
(DOCX)
