# Supplementary material for: A Unified Framework Integrating Parent-of-Origin Effects for Association Study
Source: PLoS One. 2013 Aug 26;8(8):e72208. doi: 10.1371/journal.pone.0072208 (PMC3753359; doi:10.1371/journal.pone.0072208)
Supplement: Text S1 — POE models before transformation. (DOCX) [file pone.0072208.s005.docx]

**Text S1: POE models before transformation**

We use $a_{1}$and $a_{2}$ (or $\alpha_{1}$, $\alpha_{2}$) to denote the POE, from maternal and paternal origin, respectively. We extended the usual functional (Func-Usual) model (3) to the POE functional (Func-POE) model by incorporating a POE parameter into the model, and similar steps were carried out for the usual statistical model (Stat-Usual) (5) and resulted in a POE statistical model (Stat-POE).

**Model 1: POE functional (Func-POE) model:**

Under the usual coding approach, the genotypic value $G$could be expressed as

$G=\left\{\begin{matrix} G_{11}=R \\ G_{12}=R+a_{2}+d \\ G_{21}=R+a_{1}+d \\ G_{22}=R+a_{1}+a_{2} \end{matrix}\right.$. (A1)

That is

$\left(\begin{matrix} G_{11} \\ G_{12} \\ G_{21} \\ G_{22} \end{matrix}\right)=S_{F}E_{F}=\left(\begin{matrix} 1 & 0 & 0 & 0 \\ 1 & 0 & 1 & 1 \\ 1 & 1 & 0 & 1 \\ 1 & 1 & 1 & 0 \end{matrix}\right)\left(\begin{matrix} R \\ a_{1} \\ a_{2} \\ d \end{matrix}\right)$. (A2)

The inverse is

$E_{F_{1}}=S_{F_{1}}^{-1}G$,

$\left(\begin{matrix} R \\ a_{1} \\ a_{2} \\ d \end{matrix}\right)=\left(\begin{matrix} 1 & 0 & 0 & 0 \\ -\frac{1}{2} & -\frac{1}{2} & \frac{1}{2} & \frac{1}{2} \\ -\frac{1}{2} & \frac{1}{2} & -\frac{1}{2} & \frac{1}{2} \\ -\frac{1}{2} & \frac{1}{2} & \frac{1}{2} & -\frac{1}{2} \end{matrix}\right)\left(\begin{matrix} G_{11} \\ G_{12} \\ G_{21} \\ G_{22} \end{matrix}\right)$. (A3)

Simply, we could express genotypic value $G$on the number of the paternal or maternal reference allele, as follows:

$G=R+N_{1}a_{1}+N_{2}a_{2}+\varepsilon d$. (A4)

**Model 2: POE statistical (Stat-POE) model:**

From multiple linear regression, . consists of three of the four regression parameters, $\mu$,$\alpha_{1}$,and$\alpha_{2}$.$X_{1}$ is a $n\times3$vector of $N_{1}$ and$N_{2}$information and

$X_{1}=\left(\begin{matrix} 1 & 0 & 0 \\ 1 & 0 & 1 \\ 1 & 1 & 0 \\ 1 & 1 & 1 \\ \vdots& \vdots& \vdots\\ 1 & 0 & 0 \end{matrix}\right)$. (A5)

$y$is the observed trait phenotype and $y=Z_{2}\cdot G$when those observations perfectly fit the genotypic values in ideal situation. Therefore, we could get the expression of the three parameter in , as . Additionally, the dominance effect$\delta=\frac{G_{12}+G_{21}}{2}-\frac{G_{11}+G_{22}}{2}$. After combining the coding of the additive effects and dominant effects, adjusting the coding for the intercept term$\mu$, finally we got

$E_{S_{1}}=\left(\begin{matrix} \mu\\ a_{1} \\ a_{2} \\ \delta\end{matrix}\right)=S_{S_{1}}^{-1}G=\left(\begin{matrix} p_{11} & p_{12} & p_{21} & p_{22} \\ p_{11}^{'} & p_{12}^{'} & p_{21}^{'} & p_{22}^{'} \\ p_{11}^{''} & p_{12}^{''} & p_{21}^{''} & p_{22}^{''} \\ -\frac{1}{2} & \frac{1}{2} & \frac{1}{2} & -\frac{1}{2} \end{matrix}\right)\left(\begin{matrix} G_{11} \\ G_{12} \\ G_{21} \\ G_{22} \end{matrix}\right)$. (A6)

$S_{S_{1}}^{-1}$can also be expressed as

$\left(\begin{matrix} p_{11} & p_{12} & p_{21} & p_{22} \\ -\frac{p_{11}p_{21}\overline{N}_{2}}{D} & \frac{-p_{12}p_{22}\left(1-\overline{N}_{2}\right)}{D} & \frac{p_{11}p_{21}\overline{N}_{2}}{D} & \frac{p_{12}p_{22}\left(1-\overline{N}_{2}\right)}{D} \\ -\frac{p_{11}p_{12}\overline{N}_{1}}{D} & \frac{p_{11}p_{12}\overline{N}_{1}}{D} & -\frac{p_{21}p_{22}\left(1-\overline{N}_{1}\right)}{D} & \frac{p_{21}p_{22}\left(1-\overline{N}_{1}\right)}{D} \\ -\frac{1}{2} & \frac{1}{2} & \frac{1}{2} & -\frac{1}{2} \end{matrix}\right)$. (A7)

The inverse is

$G=S_{S_{1}}E_{S_{1}}$.

It could be also expressed as

$G=\mu+\left(N_{1}-\overline{N}_{1}\right)\alpha_{1}+\left(N_{2}-\overline{N}_{2}\right)\alpha_{2}+\varepsilon\delta$, (A8)

which is equal to

$\left(\begin{matrix} G_{11} \\ G_{12} \\ G_{21} \\ G_{22} \end{matrix}\right)=S_{S_{1}}E_{S_{1}}=\left(\begin{matrix} 1 & -\overline{N}_{1} & -\overline{N}_{2} & \varepsilon_{11} \\ 1 & -\overline{N}_{1} & 1-\overline{N}_{2} & \varepsilon_{12} \\ 1 & 1-\overline{N}_{1} & -\overline{N}_{2} & \varepsilon_{21} \\ 1 & 1-\overline{N}_{1} & 1-\overline{N}_{2} & \varepsilon_{22} \end{matrix}\right)\left(\begin{matrix} \mu\\ \alpha_{1} \\ \alpha_{2} \\ \delta\end{matrix}\right)$. (A9)

This relation between the functional model and the statistical model parameters is

$\left(\begin{matrix} \mu\\ \alpha_{1} \\ \alpha_{2} \\ \delta\end{matrix}\right)=\left(\begin{matrix} 1 & \overline{N}_{1} & \overline{N}_{2} & p_{12}+p_{21} \\ 0 & 1 & 0 & p_{12}^{'}+p_{21}^{'} \\ 0 & 0 & 1 & p_{12}^{''}+p_{21}^{''} \\ 0 & 0 & 0 & 1 \end{matrix}\right)\left(\begin{matrix} R \\ a_{1} \\ a_{2} \\ d \end{matrix}\right)$. (A10)

Then, we transformed these two models to two equivalent models (10) and (13) by re-parameterization using$r_{1}=a_{1}+a_{2}$, $r_{2}=a_{2}-a_{1}$for the functional model and $\gamma_{1}=\alpha_{1}+\alpha_{2}$, $\gamma_{2}=\alpha_{2}-\alpha_{1}$ for the statistical model. These two frameworks are equivalent to some extent, whereas the transformed one is more straightforward than this original framework for nominating the overall genetic effect and POE separately.
