# Supplementary material for: A Unified Framework Integrating Parent-of-Origin Effects for Association Study
Source: PLoS One. 2013 Aug 26;8(8):e72208. doi: 10.1371/journal.pone.0072208 (PMC3753359; doi:10.1371/journal.pone.0072208)
Supplement: Text S2 — Orthogonality of the Stat-POE model before transformation. (DOCX) [file pone.0072208.s006.docx]

**Text S2 : Orthogonality of the Stat-POE model before transformation**

In the Stat-POE model, from formulation A7, we could decompose the variance of the phenotypic value $G$as

$V_{G}=Var\left[\left(N_{1}-\overline{N}_{1}\right)\alpha_{1}\right]+Var\left[\left(N_{2}-\overline{N}_{2}\right)\alpha_{2}\right]+Var\left(\varepsilon\delta\right)+2Cov\left[\left(N_{1}-\overline{N}_{1}\right)\alpha_{1},\left(N_{2}-\overline{N}_{2}\right)\alpha_{2}\right]$, (B1)

As

$Cov\left[\left(N_{1}-\overline{N}_{1}\right)\alpha_{1},\varepsilon\delta\right]=\alpha_{1}\delta Cov\left(N_{1},\varepsilon\right)=0$, (B2)

and similarly,

$Cov\left[\left(N_{2}-\overline{N}_{2}\right)\alpha_{2},\varepsilon\delta\right]=\alpha_{2}\delta Cov\left(N_{2},\varepsilon\right)=0$. (B3)

Moreover, $Var\left(\varepsilon\delta\right)=\delta^{2}Var\left(\varepsilon\right)=4p{}_{11}p_{12}p_{21}p_{22}\delta^{2}/D$. Therefore, we could express the additive and dominant variance components as

$V_{\alpha}=\alpha_{1}^{2}Var\left(N_{1}\right)+\alpha_{2}^{2}Var\left(N_{2}\right)+2\alpha_{1}\alpha_{2}Cov\left(N_{1},N_{2}\right)$. (B4)

$V_{\delta}=4p{}_{11}p_{12}p_{21}p_{22}\delta^{2}/D$. (B5)

To show the additive variance, $V_{\alpha}$could be decomposed to be two parts which are only dependent on two additive effects ($\gamma_{1}$and$\gamma_{2}$), respectively, $Cov\left(N_{1},N_{2}\right)=0$ needs to be satisfied. And as we know$Cov\left(N_{1},N_{2}\right)=E\left(N_{1}N_{2}\right)-E\left(N_{1}\right)E\left(N_{2}\right)=p_{11}p_{22}-\overline{N}_{1}\overline{N}_{2}=p_{11}p_{22}-p_{21}p_{12}$,which indeed equals to 0 if the locus is in HWE. In this way,

$V_{\alpha_{1}}=\alpha_{1}^{2}Var\left(N_{1}\right)=\alpha_{1}^{2}V_{1}$, (B6)

$V_{\alpha_{2}}=\alpha_{2}^{2}Var\left(N_{2}\right)=\alpha_{2}^{2}V_{2}$. (B7)

The two additive variance components $V_{\alpha_{1}}$ and $V_{\alpha_{2}}$are related only to the additive effects $\alpha_{1}$and $\alpha_{2}$, respectively, with one due to maternal alleles and the other due to paternal alleles. The dominant variance component $V_{\delta}$ is only related with the dominant effect $\delta$, This property of the variance component to be divided into two independent additive components and one dominant component supports the notion that the POE statistical model before transformation is orthogonal.
