# Supplementary material for: A Unified Framework Integrating Parent-of-Origin Effects for Association Study
Source: PLoS One. 2013 Aug 26;8(8):e72208. doi: 10.1371/journal.pone.0072208 (PMC3753359; doi:10.1371/journal.pone.0072208)
Supplement: Text S3 — Orthogonality of the Stat-POE model after transformation. (DOCX) [file pone.0072208.s007.docx]

**Text S3: Orthogonality of the Stat-POE model after transformation**

Except the variance component decomposition approach, the orthogonality of the Stat-POE models could also be checked by showing $X^{T}\cdot X$is a diagonal matrix in which$X$is the $n\times4$design matrix for the sample. As described in the original NOIA paper [15],

$X^{T}\cdot X=nS^{T}\cdot D\cdot S$(C1)

needs to be satisfied, where

$D=\left(\begin{matrix} p_{11} & 0 & 0 & 0 \\ 0 & p_{12} & 0 & 0 \\ 0 & 0 & p_{21} & 0 \\ 0 & 0 & 0 & p_{22} \end{matrix}\right)$. (C2)

Given that $S=\left(s_{ij}\right)$ shown in equation (5) with $s_{i1}=1$, from (C1) and (C2) we derive the criteria for orthogonality when POE incorporated as

$p_{11}s_{12}+p_{12}s_{22}+p_{21}s_{32}+p_{22}s_{42}=0$, (C3)

$p_{11}s_{13}+p_{12}s_{23}+p_{21}s_{33}+p_{22}s_{43}=0$, (C4)

$p_{11}s_{14}+p_{12}s_{24}+p_{21}s_{34}+p_{22}s_{44}=0$, (C5)

$p_{11}s_{12}s_{13}+p_{12}s_{22}s_{23}+p_{21}s_{32}s_{33}+p_{22}s_{42}s_{43}=0$, (C6)

$p_{11}s_{12}s_{14}+p_{12}s_{22}s_{24}+p_{21}s_{32}s_{34}+p_{22}s_{42}s_{44}=0$, (C7)

$p_{11}s_{13}s_{14}+p_{12}s_{23}s_{24}+p_{21}s_{33}s_{34}+p_{22}s_{43}s_{44}=0$. (C8)

Except equation (C6), all of these criteria are satisfied by $S$ in equation (5). And for equation (C6), $p_{11}s_{12}s_{13}+p_{12}s_{22}s_{23}+p_{21}s_{32}s_{33}+p_{22}s_{42}s_{43}=\frac{\left(p_{21}-p_{12}\right)\left(p_{22}-p_{11}\right)}{4}=0$when $p_{21}=p_{12}$ or $p_{11}=p_{22}$ holds true.
