# Supplementary material for: A Unified Framework Integrating Parent-of-Origin Effects for Association Study
Source: PLoS One. 2013 Aug 26;8(8):e72208. doi: 10.1371/journal.pone.0072208 (PMC3753359; doi:10.1371/journal.pone.0072208)
Supplement: Text S4 — Orthogonality of the Func-POE models. (DOCX) [file pone.0072208.s008.docx]

**Text S4: Orthogonality of the Func-POE models**

We also checked the variance decomposition of the Func-POE models before and after transformation.

For the Func-POE model before transformation, as from formulation (A4),

$V_{G}=a_{1}^{2}Var\left(N_{1}\right)+a_{2}^{2}Var\left(N_{2}\right)+Var\left(\varepsilon d\right)+a_{1}a_{2}Cov\left(N_{1},N_{2}\right)+a_{1}dCov\left(N_{1},\varepsilon\right)+a_{2}dCov\left(N_{2},\varepsilon\right)$, (D1)

where $Cov\left(N_{1},N_{2}\right)=0$under HWE which we already showed previously in Text S2, and

$Cov\left(N_{1},\varepsilon\right)=p_{21}-\left(p_{21}+p_{22}\right)\left(p_{12}+p_{21}\right)$,

$Cov\left(N_{2},\varepsilon\right)=p_{12}-\left(p_{12}+p_{22}\right)\left(p_{12}+p_{21}\right)$.

Neither of above equations was equal to 0 even when the locus was under HWE.

For the Func-POE model after transformation, as from formulation (10),

$V_{G}=\frac{r_{1}^{2}}{4}Var\left(N_{1}+N_{2}\right)+\frac{r_{2}^{2}}{4}Var\left(N_{2}-N_{1}\right)+Var\left(\varepsilon d\right)+\frac{r_{1}d}{2}Cov\left(N_{1}+N_{2},\varepsilon\right)+\frac{r_{2}d}{2}Cov\left(N_{2}-N_{1},\varepsilon\right)$,(D2)

Where $Cov\left(N_{1}+N_{2},\varepsilon\right)=\left(p_{12}+p_{21}\right)\left(p_{11}-p_{22}\right)$ is not equal to 0, and $Cov\left(N_{2}-N_{1},\varepsilon\right)=\left(p_{11}+p_{22}\right)\left(p_{12}-p_{21}\right)=0$if it is under HWE. Then

$V_{G}=\frac{r_{1}^{2}}{4}Var\left(N_{1}+N_{2}\right)+\frac{r_{2}^{2}}{4}Var\left(N_{2}-N_{1}\right)+Var\left(\varepsilon d\right)+\frac{r_{1}d}{2}Cov\left(N_{1}+N_{2},\varepsilon\right)$. (D3)

Therefore, even if the HWE assumption holds, the variance of the Func-POE models could not be expressed as the completely decomposed form as could the Stat-POE models, which confirms that the Func-POE models are not orthogonal.
