# Supplementary material for: A Unified Framework Integrating Parent-of-Origin Effects for Association Study
Source: PLoS One. 2013 Aug 26;8(8):e72208. doi: 10.1371/journal.pone.0072208 (PMC3753359; doi:10.1371/journal.pone.0072208)
Supplement: Text S5 — Test Statistics for Full and Reduced One-Locus Models. (DOCX) [file pone.0072208.s009.docx]

**Text S5: Test Statistics for Full and Reduced One-Locus Models**

The Wald test statistic is , where

The $n$ rows of matrix$Z$represent the corresponding genotype for individual $i$. And $X=Z\cdot S$.

$Z=\left(\begin{matrix} 1 & 0 & 0 \\ \vdots& \vdots& \vdots\\ 0 & 1 & 0 \\ \vdots& \vdots& \vdots\\ 0 & 0 & 1 \\ \vdots& \vdots& \vdots\end{matrix}\right)$, and $Z^{'}Z=n\left(\begin{matrix} p_{11} & 0 & 0 \\ 0 & p_{12} & 0 \\ 0 & 0 & p_{22} \end{matrix}\right)$

$$X^{'}X=\left(ZS\right)^{'}ZS=S^{'}Z^{'}ZS$$

We recall that,$\overline{N}=p_{12}+2p_{22}$, $V=p_{12}+4p_{22}-\overline{N}^{2}=4p_{11}p_{22}+p_{11}p_{12}+p_{12}p_{22}$.

**For NOIA statistical model with no dominance component modeled,**

$S=\left(\begin{matrix} 1 & -\overline{N} \\ 1 & 1-\overline{N} \\ 1 & 2-\overline{N} \end{matrix}\right)$

$$X^{'}X=S^{'}Z^{'}ZS=\left(\begin{matrix} 1 & 1 & 1 \\ -\overline{N} & 1-\overline{N} & 2-\overline{N} \end{matrix}\right)\cdot n\left(\begin{matrix} p_{11} & 0 & 0 \\ 0 & p_{12} & 0 \\ 0 & 0 & p_{22} \end{matrix}\right)\cdot\left(\begin{matrix} 1 & -\overline{N} \\ 1 & 1-\overline{N} \\ 1 & 2-\overline{N} \end{matrix}\right)=n\left(\begin{matrix} 1 & 0 \\ 0 & V \end{matrix}\right)$$

$\det\left(X^{'}X\right)=nV$

$\left(X^{'}X\right)^{-1}=\frac{1}{nV}\left(\begin{matrix} V & 0 \\ 0 & 1 \end{matrix}\right)$

Then

**For NOIA statistical model with dominance component,**

$S_{S}=\left(\begin{matrix} 1 & -\overline{N} & -\frac{2p_{12}p_{22}}{V} \\ 1 & 1-\overline{N} & \frac{4p_{11}p_{22}}{V} \\ 1 & 2-\overline{N} & -\frac{2p_{11}p_{12}}{V} \end{matrix}\right)$

$$\begin{matrix} X^{'}X=S^{'}Z^{'}ZS=n\left(\begin{matrix} 1 & 1 & 1 \\ -\overline{N} & 1-\overline{N} & 2-\overline{N} \\ -\frac{2p_{12}p_{22}}{V} & \frac{4p_{11}p_{22}}{V} & -\frac{2p_{11}p_{12}}{V} \end{matrix}\right)\cdot\left(\begin{matrix} p_{11} & 0 & 0 \\ 0 & p_{12} & 0 \\ 0 & 0 & p_{22} \end{matrix}\right)\cdot\left(\begin{matrix} 1 & -\overline{N} & -\frac{2p_{12}p_{22}}{V} \\ 1 & 1-\overline{N} & \frac{4p_{11}p_{22}}{V} \\ 1 & 2-\overline{N} & -\frac{2p_{11}p_{12}}{V} \end{matrix}\right) \\ =n\left(\begin{matrix} 1 & 0 & 0 \\ 0 & V & 0 \\ 0 & 0 & \frac{4p_{11}p_{12}p_{22}}{V} \end{matrix}\right) \end{matrix}$$

$\det\left(X^{'}X\right)=4np_{11}p_{12}p_{22}$

$$\left(X^{'}X\right)^{-1}=\frac{1}{4np_{11}p_{12}p_{22}}\left(\begin{matrix} 4p_{11}p_{12}p_{22} & 0 & 0 \\ 0 & \frac{4p_{11}p_{12}p_{22}}{V} & 0 \\ 0 & 0 & V \end{matrix}\right)$$

Then we got the test statistic for the NOIA one-locus full model as follows:

.

**For usual functional model with no dominance component modeled,**

$S=\left(\begin{matrix} 1 & 0 \\ 1 & 1 \\ 1 & 2 \end{matrix}\right)$

$$X^{'}X=S^{'}Z^{'}ZS=n\cdot\left(\begin{matrix} 1 & 1 & 1 \\ 0 & 1 & 2 \end{matrix}\right)\cdot\left(\begin{matrix} p_{11} & 0 & 0 \\ 0 & p_{12} & 0 \\ 0 & 0 & p_{22} \end{matrix}\right)\cdot\left(\begin{matrix} 1 & 0 \\ 1 & 1 \\ 1 & 2 \end{matrix}\right)=n\left(\begin{matrix} 1 & \overline{N} \\ \overline{N} & \overline{N}+2p_{22} \end{matrix}\right)$$

$\det\left(X^{'}X\right)=nV$

$$\left(X^{'}X\right)^{-1}=\frac{1}{nV}\left(\begin{matrix} \overline{N}+2p_{22} & -\overline{N} \\ -\overline{N} & 1 \end{matrix}\right)$$

It results in the same test statistic as the NOIA model for the additive effect estimating.

**For usual functional model with dominance component modeled,**

$$X^{'}X=S_{F}^{'}Z^{'}ZS_{F}=n\cdot\left(\begin{matrix} 1 & 1 & 1 \\ 0 & 1 & 2 \\ 0 & 1 & 0 \end{matrix}\right)\cdot\left(\begin{matrix} p_{11} & 0 & 0 \\ 0 & p_{12} & 0 \\ 0 & 0 & p_{22} \end{matrix}\right)\cdot\left(\begin{matrix} 1 & 0 & 0 \\ 1 & 1 & 1 \\ 1 & 2 & 0 \end{matrix}\right)=n\left(\begin{matrix} 1 & \overline{N} & p_{12} \\ \overline{N} & p_{12}+4p_{22} & p_{12} \\ p_{12} & p_{12} & p_{12} \end{matrix}\right)$$

$\det\left(X^{'}X\right)=4np_{11}p_{12}p_{22}$

$$\left(X^{'}X\right)^{-1}=\frac{1}{4np_{11}p_{12}p_{22}}\left(\begin{matrix} 4p_{12}p_{22} & -2p_{12}p_{22} & -2p_{12}p_{22} \\ -2p_{12}p_{22} & p_{12}\left(1-p_{12}\right) & -p_{12}\left(1-\overline{N}\right) \\ -2p_{12}p_{22} & -p_{12}\left(1-\overline{N}\right) & V \end{matrix}\right)$$

From the second row of the matrix, we observe that the test statistic for the additive effect is different with those of the previous three models. And the test statistic z is usually smaller.
