# Supplementary material for: A Unified Framework Integrating Parent-of-Origin Effects for Association Study
Source: PLoS One. 2013 Aug 26;8(8):e72208. doi: 10.1371/journal.pone.0072208 (PMC3753359; doi:10.1371/journal.pone.0072208)
Supplement: Text S6 — R coding of Stat-POE and Func-POE models. (DOCX) [file pone.0072208.s010.docx]

**Text S6: R coding of Stat-POE and Func-POE models**

**For the Stat-POE model:**

dat<-read.table("inputfile1",header=F,sep = " ") #input genotype data

dat_T<-read.table("inputfile2",header=F,sep = " ") #input phenotype data

L<-matrix(NA,MKN,8) #MKN as total marker number

colnames(L)<-c("R.coef","a1.coef","a2.coef","d.coef","R.pva","a1.pva","a2.pva","d.pva")

for(i in 1:MKN) {

Ss<-matrix(NA,4,4)

V<-matrix(NA,$SAMPN,4)

n11<-length(which(dat[,i]==1)) # for genotype 11

n12<-length(which(dat[,i]==2)) # for genotype 12

n21<-length(which(dat[,i]==3)) # for genotype 21

n22<-length(which(dat[,i]==4)) # for genotype 22

p11<-n11/$SAMPN

p12<-n12/$SAMPN

p21<-n21/$SAMPN

p22<-n22/$SAMPN

N1<-p21+p22

N2<-p12+p22

detA<-p12*p21*p22+p11*p21*p22+p11*p12*p22+p11*p12*p21

Ss[,1]<-1

Ss[1,2]<--(N1+N2)*(0.5)

Ss[2,2]<-0.5-(N1+N2)*(0.5)

Ss[3,2]<-0.5-(N1+N2)*(0.5)

Ss[4,2]<-1-(N1+N2)*(0.5)

Ss[c(1,4),3]<-(N1-N2)*0.5

Ss[2,3]<-0.5+(N1-N2)*0.5

Ss[3,3]<--0.5+(N1-N2)*0.5

Ss[1,4]<-(-2)*p12*p21*p22/detA

Ss[2,4]<-2*p11*p21*p22/detA

Ss[3,4]<-2*p11*p12*p22/detA

Ss[4,4]<-(-2)*p12*p21*p11/detA

V[which(dat[,i]==1),]<-matrix(rep(Ss[1,],n11),nrow=n11,byrow=TRUE)

V[which(dat[,i]==2),]<-matrix(rep(Ss[2,],n12),nrow=n12,byrow=TRUE)

V[which(dat[,i]==3),]<-matrix(rep(Ss[3,],n21),nrow=n21,byrow=TRUE)

V[which(dat[,i]==4),]<-matrix(rep(Ss[4,],n22),nrow=n22,byrow=TRUE)

regdat<-data.frame(cbind(dat_T[,i],V[,2:4]))

fit_logit<-lm(X1~X2+X3+X4,data=regdat)

SMR<-summary(fit_logit)

for(j in 1:4){

L[i,j]<-SMR$coef[j,1]

L[i,j+4]<-SMR$coef[j,4]

}

}

write.table(L,"StatPOE_output.txt",row.names=FALSE,col.names=TRUE)

**For the Func-POE model:**

for(i in 1:MKN) {

N1<-length(which(dat[,i]==1))

N2<-length(which(dat[,i]==2))

N3<-length(which(dat[,i]==3))

N4<-length(which(dat[,i]==4))

a1<-dat[,i]

a2<-dat[,i]

d<-dat[,i]

a1[which(dat[,i]==1)]<-c(rep(0,N1))

a1[which(dat[,i]==4)]<-c(rep(1,N4))

a1[which(dat[,i]==2|dat[,i]==3)]<-c(rep(0.5,N2+N3))

a2[which(dat[,i]==1|dat[,i]==4)]<-c(rep(0,N1+N4))

a2[which(dat[,i]==2)]<-c(rep(0.5,N2))

a2[which(dat[,i]==3)]<-c(rep(-0.5,N3))

d[which(dat[,i]==2|dat[,i]==3)]<-c(rep(1,N3+N2))

d[which(dat[,i]==1|dat[,i]==4)]<-c(rep(0,N1+N4))

regdat<-data.frame(cbind(dat_T[,i],a1,a2,d))

fit_logit<-lm(V1~a1+a2+d,data=regdat)

SMR<-summary(fit_logit)

for(j in 1:4){

L[i,j]<-SMR$coef[j,1]

L[i,j+4]<-SMR$coef[j,4]

}

}

write.table(L,"FuncPOE_output.txt",row.names=FALSE,col.names=TRUE)
